# Supplementary material for: Energetics of the Transmembrane Peptide Sorting by Hydrophobic Mismatch
Source: J Phys Chem Lett. 2024 May 13;15(20):5344–9. doi: 10.1021/acs.jpclett.4c00651 (PMC11129306; doi:10.1021/acs.jpclett.4c00651)
Supplement: Supplementary file 2 — jz4c00651_si_002.pdf [file jz4c00651_si_002.pdf]

jz-2024-006519.R1

Name: Peer Review Information for "Energetics of the Transmembrane Peptide Sorting by Hydrophobic Mismatch"

First Round of Reviewer Comments

Reviewer: 1

Comments to the Author

The authors' work on using molecular dynamics simulations to study hydrophobic mismatch in lipid membranes and its impact on peptide localization and function is commendable. They introduced a "novel" method for analyzing peptide behavior in response to membrane thickness gradients, a critical factor in understanding the role of hydrophobic mismatch. Their findings, particularly the analysis of 2-dimensional free energy profiles to determine the contributions of sorting and tilting mechanisms, offer valuable insights into the thermally accessible regions of these processes. This approach has significant implications for studying other biological membrane systems affected by hydrophobic mismatch or membrane thickness variations.

However, the authors' main emphasis on the novelty of their method completely overlooks the broader context of research on membrane thickness gradients and their effects on peptide and lipid sorting. The work cited in the provided source (https://www.frontiersin.org/journals/physiology/articles/10.3389/fphys.2020.00250/full) demonstrates that similar methods have been developed to study the sorting of membrane lipids and surface-binding peptides in response to membrane thinning. This study, focusing on the amphipathic lipid packing sensor (ALPS) motif, uses a thinning protocol to induce lipid and protein sorting, aligning with the authors' approach but with a focus on membrane defect/curvature sensing.

Given this, it would be more appropriate for the authors to highlight their novel findings within the context of hydrophobic mismatch, rather than claiming novelty and originality for their method. Additionally, acknowledging and citing earlier works that introduced similar approaches, discussing the differences therein, and comparing their results with those of the cited study would provide a more comprehensive and credible view of their contributions. This approach not only strengthens their argument for the impact of hydrophobic mismatch but also contributes to the broader scientific discourse on membrane biology and protein sorting.

Reviewer: 2

Comments to the Author

The authors present a method for simulating sorting of peptides by membrane thickness (an important effect in cell biology), and illustrate it using well-done simulations of different length polyleucine transmembrane helices. This is nice work, though requires some revisions before publication.

The present method is very much like the "Binary Bilayer System (BBS) proposed by Park and Im (Chem. Theory Comput. 2018, 14, 2829−2833). A BBS was then used to calculate the hydrophobic mismatch and free energy of transfer of gramicidin A between DMPC and DLPC (Park et al (J. Chem. Theory Comput. 2019, 15, 6491−6503). These papers should be cited and put in the context of the present method.

I don't believe that the calculations distinguished systematic and dynamic tilt. Even if the peptide is on average aligned along the bilayer normal (no systematic tilt), it will, on average show a tilt because of "wobble". A peptide with systematic tilt (not, on average, aligned along the normal, like gel phase lipid chains) will also have wobble. This might resolve the differences of Leu23 and Leu27 with the other peptides. These ideas are related to those discussed by Kim and Im (ref 12).

Page 2. It is stated that the lipid types are maintained in their regions by flat bottomed potentials and Fig 1B is referenced. The reference to Fig 1B is a little misleading, as I expected to see a sketch of the reference potentials, including their functional form. I suggest the such a figure be included in the SI (perhaps as an additional panel in Fig S1), and referenced along with Fig 1B in the main text.

Page 2, col 2, last line. It would reasonable to point to Figure S3 regarding the trajectory of Leu29 initialized from the thin membrane (where it does not migrate to the thick region).

Reviewer: 3

Comments to the Author

In the manuscript titled "Energetics of the Transmembrane Peptide Sorting by Hydrophobic Mismatch", the authors performs a systematic study on the response of transmembrane helices to hydrophobic mismatch, when embedded in lipid bilayers of different thicknesses. To this aims, the authors perform a large number of biased and unbiased coarse-grained molecular dynamics simulations, investigating specifically the contributions of lateral sorting and peptide tilting.

Hydrophobic mismatch between transmembrane proteins and lipid bilayers is an interesting topic that deserves attention. The authors tackle this problem by considering the mismatch between simple helical peptides and a membrane that presents a thickness gradient. The well-described interplay between lateral sorting and peptide tilting represents a first step towards the study of hydrophobic mismatch in more complex membrane proteins. In addition, the authors develop and employ an interesting membrane model, based on the MARTINI 2.2 force field, for the simulation of a thickness gradient within a lipid bilayer.

The manuscript is well written and the study is well designed. However, I have a few comments, as described below.

- The authors state that "We first studied whether the thickness gradient sorts the peptides, or whether alternative mechanisms—such as peptide tilt or membrane deformation— dominate". However, it is still not clear whether membrane deformations play a role in the observed results. It would be useful if the authors could comment on this, investigating if and how the membrane responds to the MM and therefore affects, for instance, the measured tilt angles.

- From figures 2 and 3A, it seems that the center of mass position of the peptide during lateral sorting in the bilayer tends to remain trapped within some specific intervals along the x-coordinate. This happens also when considering one specific peptide length, such as Leu19, where two separate distributions are clearly observed. In general, not only these densely populated intervals appear equidistant, but they also seem to coincide with the interfaces between adjacent membrane patches. The authors should make sure that there is no effect of this type. In addition, the position of the shaded areas in the plots of figure 1 and figure S1 should be consistent.

Minor comments:

- Page 2, line 37: Given the important changes introduced in the most recent versions of the Martini force field, the version that has been employed should be specified in the main text when the Martini force field is mentioned for the first time.

- Page 2, line 40: I would describe the thickness profile as “constant”, in place of “permanent”.

Author's Response to Peer Review Comments:

Rebuttal

Reviewer 1

Reviewer comment:
The authors' work on using molecular dynamics simulations to study hydrophobic mismatch in lipid membranes and its impact on peptide localization and function is commendable. They introduced a "novel" method for analyzing peptide behavior in response to membrane thickness gradients, a critical factor in understanding the role of hydrophobic mismatch. Their findings, particularly the analysis of 2-dimensional free energy profiles to determine the contributions of sorting and tilting mechanisms, offer valuable insights into the thermally accessible regions of these processes. This approach has significant implications for studying other biological membrane systems affected by hydrophobic mismatch or membrane thickness variations.

Author reply:
We thank the reviewer for encouraging feedback.

Reviewer comment:
However, the authors' main emphasis on the novelty of their method completely overlooks the broader context of research on membrane thickness gradients and their effects on peptide and lipid sorting. The work cited in the provided source (https://www.frontiersin.org/journals/physiology/articles/10.3389/fphys.2020.00250/full) demonstrates that similar methods have been developed to study the sorting of membrane lipids and surface-binding peptides in response to membrane thinning. This study, focusing on the amphipathic lipid packing sensor (ALPS) motif, uses a thinning protocol to induce lipid and protein sorting, aligning with the authors' approach but with a focus on membrane defect/curvature sensing.

Given this, it would be more appropriate for the authors to highlight their novel findings within the context of hydrophobic mismatch, rather than claiming novelty and originality for their method. Additionally, acknowledging and citing earlier works that introduced similar approaches, discussing the differences therein, and comparing their results with those of the cited study would provide a more comprehensive and credible view of their contributions. This

approach not only strengthens their argument for the impact of hydrophobic mismatch but also contributes to the broader scientific discourse on membrane biology and protein sorting.

Author reply:
We have indeed not cited this work simply as we were unaware of its existence. This has been corrected in the revised version of the manuscript, where we have added the reference and also a small description of the differences with our new approach.

However, we also think that there are some features in our setup that justify the use of the word "*novel*". With the flat-bottom restraints, we are able to induce the thickness gradient with a fairly constant lipid density along the gradient, unlike with the method referred to by the Reviewer. Hence, our method captures the membrane in the native, tensionless state. Secondly, our approach does not require a modified version of GROMACS.

Reviewer 2

Reviewer comment:
The authors present a method for simulating sorting of peptides by membrane thickness (an important effect in cell biology), and illustrate it using well-done simulations of different length polyleucine transmembrane helices. This is nice work, though requires some revisions before publication.

Author reply:
We thank the Reviewer for their kind words.

Reviewer comment:
The present method is very much like the "Binary Bilayer System (BBS) proposed by Park and Im (Chem. Theory Comput. 2018, 14, 2829−2833). A BBS was then used to calculate the hydrophobic mismatch and free energy of transfer of gramicidin A between DMPC and DLPC (Park et al (J. Chem. Theory Comput. 2019, 15, 6491−6503). These papers should be cited and put in the context of the present method.

Author reply:
We thank the reviewer for pointing out these works, which should indeed be cited. Still, we do not feel that they are 'very much like', since our approach provides a smooth thickness gradient, which induces a harmonic force position-dependent force on the peptides, unlike the biphasic system described in the suggested papers. We have added the references to the revised manuscript, along with a short description on the key differences to our approach.

Reviewer comment:

I don’t believe that the calculations distinguished systematic and dynamic tilt. Even if the peptide is on average aligned along the bilayer normal (no systematic tilt), it will, on average show a tilt because of “wobble”. A peptide with systematic tilt (not, on average, aligned along the normal, like gel phase lipid chains) will also have wobble. This might resolve the differences of Leu23 and Leu27 with the other peptides. These ideas are related to those discussed by Kim and Im (ref 12).

Author reply:
We are not certain that we understood this question. The peptides show a tilt simply due to an entropic contribution (there are many more states available for tilted conformations than the 1(!) state for the perfectly upright conformation. This contribution is inherently included in the analysis we perform, and it shows the difference for Leu23 and Leu27.

Still, another potential factor that could lead to directional tilt is the thickness gradient. However, we believed its effect to be minor, since the local thickness variation is minimal. We quantified this effect by extracting the azimuthal angles of the vector spanning the peptide. We compared the obtained distribution against a uniform distribution using one-sample Kolmogorov–Smirnov tests. As demonstrated in the SI and commented in the revised manuscript, these tests revealed that there was no systematic orientational tilt preference for any of the peptides.

Reviewer comment:
Page 2. It is stated that the lipid types are maintained in their regions by flat bottomed potentials and Fig 1B is referenced. The reference to Fig 1B is a little misleading, as I expected to see a sketch of the reference potentials, including their functional form. I suggest the such a figure be included in the SI (perhaps as an additional panel in Fig S1), and referenced along with Fig 1B in the main text.

Author reply:
This is a great suggestion, and such a panel has been added to the revised Fig. S1 in the SI, along with the density of the different lipid types.

Reviewer comment:
Page 2, col 2, last line. It would reasonable to point to Figure S3 regarding the trajectory of Leu29 initialized from the thin membrane (where it does not migrate to the thick region).

Author reply:
We have added this reference to both the text and the caption in which this issue was mentioned.

Reviewer 3

Reviewer comment:
In the manuscript titled "Energetics of the Transmembrane Peptide Sorting by Hydrophobic Mismatch", the authors performs a systematic study on the response of transmembrane helices to hydrophobic mismatch, when embedded in lipid bilayers of different thicknesses. To this aims, the authors perform a large number of biased and unbiased coarse-grained molecular dynamics simulations, investigating specifically the contributions of lateral sorting and peptide tilting.

Hydrophobic mismatch between transmembrane proteins and lipid bilayers is an interesting topic that deserves attention. The authors tackle this problem by considering the mismatch between simple helical peptides and a membrane that presents a thickness gradient. The well-described interplay between lateral sorting and peptide tilting represents a first step towards the study of hydrophobic mismatch in more complex membrane proteins. In addition, the authors develop and employ an interesting membrane model, based on the MARTINI 2.2 force field, for the simulation of a thickness gradient within a lipid bilayer.

The manuscript is well written and the study is well designed. However, I have a few comments, as described below.

Author reply:
We thank the Reviewer for their constructive feedback.

Reviewer comment:
- The authors state that "We first studied whether the thickness gradient sorts the peptides, or whether alternative mechanisms—such as peptide tilt or membrane deformation—dominate". However, it is still not clear whether membrane deformations play a role in the observed results. It would be useful if the authors could comment on this, investigating if and how the membrane responds to the MM and therefore affects, for instance, the measured tilt angles.

Author reply:
We are grateful that the Reviewer caught this inconsistency. We have now analyzed the membrane thickness perturbations induced by the peptides, and our simulations revealed that the membrane thickening ability of even long peptides is meager (~0.1 nm), the short peptides can induce significant thinning of up to ~0.5 nm in the thick regions of the membrane. These results are presented in Figs. 4A in the revised manuscript and Fig. S7 in the revised SI.

Reviewer comment:
- From figures 2 and 3A, it seems that the center of mass position of the peptide during lateral sorting in the bilayer tends to remain trapped within some specific intervals along the x-

coordinate. This happens also when considering one specific peptide length, such as Leu19, where two separate distributions are clearly observed. In general, not only these densely populated intervals appear equidistant, but they also seem to coincide with the interfaces between adjacent membrane patches. The authors should make sure that there is no effect of this type.

Author reply:
We indeed observe some bias of the peptides towards the regions with no overlap of the lipid species. We have tested different degrees of overlap between the neighbouring phases, and the one used in the production simulations provides by far the smoothest thickness gradient.

We have now also simulated our membrane setup with 5 lipids of each type and in both leaflets released from flat-bottom restraints, hence allowing them to freely sample the membrane plane. We again observe the small yet systematic repulsive bias at the mixed lipid regions (Fig. S1F in the revised manuscript). Similar boundary effects seem to also haunt earlier works with (forced) multi-phase systems (see added discussion in the revised manuscript).

We also further characterised our simulation setup, and noticed nothing odd in the densities of the different lipid types that could explain this bias. Hence, it could be an inherent limitation of the coarse-grained approach with limited resolution in describing the acyl chains. This was already mentioned in the original manuscript, and we have now extended the discussion in the revised manuscript.

Reviewer comment:
In addition, the position of the shaded areas in the plots of figure 1 and figure S1 should be consistent.

Author reply:
This was a good observation, and the shading in Fig. 1 has now been corrected.

Reviewer comment:
- Page 2, line 37: Given the important changes introduced in the most recent versions of the Martini force field, the version that has been employed should be specified in the main text when the Martini force field is mentioned for the first time.

Author reply:
We have added the Martini version to the text. Unfortunately the Martini 3 lipidome is not available yet, but the transformation of our protocol from Martini 2 to Martini 3 should be straightforward.

Reviewer comment:
- Page 2, line 40: I would describe the thickness profile as “constant”, in place of “permanent”.

Author reply:
We have made this suggested change.

jz-2024-006519.R2

Name: Peer Review Information for "Energetics of the Transmembrane Peptide Sorting by Hydrophobic Mismatch"

Second Round of Reviewer Comments

Reviewer: 2

Comments to the Author

The authors have revised their ms in accord with my comments.

Reviewer: 3

Comments to the Author

The authors have properly addressed the issues I had pointed out.

Reviewer: 1

Comments to the Author

The authors addressed my main concerns/comments. I only have a few minor comments:

In the paper, the authors highlight the versatility of their setup for studying various membrane-embedded objects, including lipids, peptides, proteins, and their complexes. However, a critical aspect of their methodology warrants further clarification.

The authors state, "This indicates that our setup can be efficiently used to study the sorting of lipids, peptides, proteins and their complexes, and other membrane-embedded objects." While this assertion underscores the broad applicability of their approach, it overlooks the inherent limitations of gradient methods in studying proteins, particularly those of significant size, such as membrane channels.

For efficient sorting of membrane-embedded objects within the constraints of simulation time scales, it is crucial to maintain sufficiently strong gradients while ensuring the membrane systems are not excessively large. This balance is essential to accurately represent the sorting process and to avoid computational inefficiencies. However, the change in gradient must be negligible at the scale of the protein being studied. This limitation is crucial for accurately representing the behavior of larger proteins within the simulation environment.

To enhance the clarity of their statement, the authors could specify that while their setup is highly adaptable, it is optimized for smaller proteins and may face challenges when applied to larger structures. This nuanced perspective would provide a more accurate representation of the setup's capabilities and limitations.

Author's Response to Peer Review Comments:

Rebuttal

Reviewer: 1

Reviewer comment:

In the paper, the authors highlight the versatility of their setup for studying various membrane-embedded objects, including lipids, peptides, proteins, and their complexes. However, a critical aspect of their methodology warrants further clarification.

The authors state, "This indicates that our setup can be efficiently used to study the sorting of lipids, peptides, proteins and their complexes, and other membrane-embedded objects." While this assertion underscores the broad applicability of their approach, it overlooks the inherent limitations of gradient methods in studying proteins, particularly those of significant size, such as membrane channels.

For efficient sorting of membrane-embedded objects within the constraints of simulation time scales, it is crucial to maintain sufficiently strong gradients while ensuring the membrane systems are not excessively large. This balance is essential to accurately represent the sorting process and to avoid computational inefficiencies. However, the change in gradient must be negligible at the

scale of the protein being studied. This limitation is crucial for accurately representing the behavior of larger proteins within the simulation environment.

To enhance the clarity of their statement, the authors could specify that while their setup is highly adaptable, it is optimized for smaller proteins and may face challenges when applied to larger structures. This nuanced perspective would provide a more accurate representation of the setup's capabilities and limitations.

Author reply:

The Reviewer is correct in that the inclusion of very large proteins or their complexes in the present setup might be problematic, as the protein itself would span a large range of the membrane and hence a large thickness difference. In our opinion this is not a limitation for commonly studied proteins, ranging from single-pass TM peptides to G protein-coupled receptors and ion channels. For larger ones, the phases in our setup can be extended, and this might require slight optimization of the overlap between neighbouring phases, as well as the extension of the membrane size in the direction perpendicular to the gradient. Still, at least in Martini such simulations are well feasible, and can be sampled at a rate of multiple microseconds per day using modern day computing hardware.

We have added this possibility of extension for larger proteins in the revised manuscript.
